# Quantifying the risk of sexual dysfunction in men treated with pelvic radiation therapy for locally advanced rectal cancer

**DOI:** 10.3389/fonc.2026.1813135

**Published:** 2026-06-17

**Authors:** Kush H. Patel, Neal Kim, Kathryn R. Tringale, Lily Boe, Marsha Reyngold, Abraham Jing-Ching Wu, Marsha Reyngold, Paul Romesser, John Cuaron, Emmanouil Pappou, Maliha Nusrat, John P. Mulhall, Carla Hajj

**Affiliations:** 1 Department of Radiation Oncology, Memorial Sloan Kettering Cancer Center, New York, NY, United States; 2 Department of Medical Physics, Memorial Sloan Kettering Cancer Center, New York, NY, United States; 3 Department of Epidemiology and Biostatistics, Memorial Sloan Kettering Cancer Center, New York, NY, United States; 4 Department of Surgery, Memorial Sloan Kettering Cancer Center, New York, NY, United States; 5 Sexual & Reproductive Medicine Program, Urology Service, Memorial Sloan Kettering Cancer Center, New York, NY, United States

**Keywords:** ejaculatory dysfunction (EjD), erectile dysfunction (ED), radiation therapy, rectal cancer, sexual dysfunction, survivorship, young males

## Abstract

**Purpose:**

As the incidence of stage II-III locally advanced rectal cancer (LARC) rises in younger patients, we sought to investigate the risk of sexual dysfunction following radiation therapy (RT) in younger men.

**Methods:**

Men ≤50 years old diagnosed with LARC between 1995–2019 at a single institution were retrospectively evaluated. Primary outcomes of erectile dysfunction (ED) and ejaculatory dysfunction (EjD) were defined via ICD codes and clinical evaluation. Cumulative incidences were calculated with death as a competing risk. Subdistribution hazard ratios from competing risk regression models were used. Dosimetric parameters to organs at risk were extracted to suggest dose constraints to prevent sexual dysfunction.

**Results:**

The study included 429 men: 350 received concurrent chemoradiation therapy (CXRT) as part of their treatment, and 79 did not receive RT. The two groups were similar with respect to age at diagnosis, stage, and medical comorbidities associated with sexual dysfunction (p>0.05). With a median follow-up of 6.3 years, the 5-year cumulative incidence of ED in the CXRT group was 25% (95%CI 20-30%) compared to 3.9% (95%CI 1.0-10%) in the no RT group (p<0.001). On multivariable analysis, CXRT maintained statistical significance as an independent risk factor for ED (HR 3.55, 95% CI 1.34-9.44, p=0.011). Within the CXRT group, inguinal coverage in the treatment fields was an independent risk factor for ED (HR 2.38, 95% CI 1.30-4.34, p=0.005), and greater distance from the anal verge had a protective effect. No difference was found in EjD between the 2 groups.

**Conclusion:**

CXRT independently increases risk of ED but not EjD in patients treated for rectal cancer. Technique and target location may impact sexual dysfunction, possibly due to greater radiation dose to the genitalia. These findings may aid in establishing new constraints and promote counseling of young male patients on risks of sexual dysfunction.

## Introduction

Colorectal cancer (CRC) is the second leading cause of cancer death in the United States. The overall incidence of CRC has declined between 1-4% annually during the 21^st^ century, but this has been opposed by a 1-2% increase in patients ≤55 years old. The proportion of cases among those ≤55 years old increased from 11% to 20% between 1995 to 2019, and CRC is now the leading cause of cancer death in males younger than 50 years old (1). With younger men being diagnosed with CRC it is crucial to emphasize survivorship. Therefore, sexual function is an important quality of life factor that should be considered when treating younger patients.

Locally advanced rectal cancer (LARC) is defined as American Joint Commission on Cancer (AJCC) stage 2 (T3-4, node negative) and stage 3 (node positive) rectal cancer. The treatment for LARC involves a multimodality approach consisting of surgery, chemotherapy, and pelvic radiation therapy (RT) (2). Each treatment modality poses a potential risk of negatively impacting reproductive and sexual health. While the addition of RT in treatment has shown benefit in the reduction of local recurrence rates, the risks of neurovascular damage, and vascular damage to erectile bodies (corporal bodies) remain unclear (3). These risks may be further increased in patients with lower-lying rectal tumors who may require inguinal nodes to be included into their treatment plans.

Sexual toxicity from radiation for prostate cancer is well studied and documented. These studies have helped to identify key anatomy which contributes to sexual dysfunction when exposed to certain doses of radiation. These organs and regions include the penile bulb, corpora cavernosa, neurovascular bundles, and crura (4–6). Thanks to these studies active efforts are made to limit dose to these organs when planning radiation treatment for prostate cancer.

Compared to prostate cancer, previous studies investigating sexual dysfunction following rectal cancer treatment have been much more limited. Similarly, these studies have shown addition of RT for LARC treatment has been associated with decreases in sexual desire, frequency of intercourse, possibility to reach an orgasm, satisfaction, and with higher rates of erectile dysfunction (ED) (7–12). Despite prior studies suggesting risks of sexual dysfunction from rectal cancer treatment, there appears to be a lack of transparency between oncology providers and patients about how treatments can affect sex life (13). While these studies have explored the topics of sexual dysfunction after RT, the variation in definitions of sexual dysfunction, short follow-up, and pairing of colon and rectal cancers together, makes interpretation of these studies difficult. Understanding the risks of sexual dysfunction following pelvic RT, particularly in long-term survivorship, is critical to selecting the optimal treatment approach on an individualized level.

We aimed to design a large sample study of young rectal cancer patients to assess the independent risk of RT on ED and ejaculatory dysfunction (EjD).

## Methods

### Patient population

This retrospective study included men diagnosed with locally advanced rectal cancer at age ≤50 years from 1995–2019 at a single private non-profit institution. Men age ≤50 were selected to minimize impact of biological age on sexual health as ED in men after age 50 is common (14). Demographic characteristics (e.g., race, age at diagnosis) and known risk factors for sexual dysfunction (e.g., hypertension, dyslipidemia, diabetes, coronary artery disease (CAD), cerebrovascular accident, body mass index, tobacco use, depression) were collected. The institutional review board approved this study prior to data collection and analysis (IRB16-370).

### Treatment cohorts

Patients were grouped into two cohorts based on whether they had received pelvic RT or not as part of their multimodality treatment. Patients who received RT also received concurrent chemotherapy comprise the “CXRT” group, and the remaining patients comprise the “no RT” group. Patients in both groups were treated according to institutional guidelines with adjuvant chemotherapy. The treatment approach to LARC evolved over the course of the study period, so year of treatment before or after 2004 was noted since that was when preoperative chemoradiation was established as standard of care (15). Other treatment variables such as surgery, use of concurrent chemo, RT delivery technique, and prescription dose were collected. RT delivery techniques were grouped into two classifications: 3D conformal and conventional RT were classified as “3D RT”, and static intensity-modulated RT (IMRT) and volumetric modulated arc therapy (VMAT) were classified as “IMRT”. Patients were excluded if they had received prior pelvic RT or if their treatment was for recurrent disease (defined as having started chemotherapy or RT more than one year after diagnosis of locally advanced disease).

### Definition of sexual dysfunction

Sexual dysfunction was further classified by ED and/or EjD based on ICD9/10 codes or encounters at our institution’s sexual health clinic and visit dispositions. ED included diagnoses related to ‘impotence’ and ‘male erectile dysfunction’ and EjD included diagnoses related to retrograde ejaculation, painful ejaculation, premature ejaculation, reduced ejaculation, and anejaculation. Development of one category of sexual dysfunction did not preclude capturing the other category of dysfunction. Given the purpose of this study was to identify RT-related sexual dysfunction, patients with prior documented dysfunction or those on androgen deprivation therapy (ADT) for any reason were excluded from our dataset.

### RT dosimetry

Of 350 patients who received RT, 182 had radiation treatment plans available in our planning system. Organs at risk (OARs) were contoured on the planning CT scans and doses to these structures were recorded. The dosimetric parameters extracted included the maximum, mean, D1cc, D3cc, and D5cc doses as well as the equivalent doses in 2 Gy fractions (EQD2). OARs of particular interest were the testes, penile bulb, corpus spongiosum, corpus cavernosum, and glans penis. These dosimetric parameters were then used to find a value, a cutpoint, at which beyond the risk for sexual function was greatest.

A descriptive guide of OARs of interest contouring is included in **Supplementary Table 1**, followed by a visual representation in **Supplementary Figure 1**.

### Statistical methods

Descriptive statistics were used to summarize patient clinicodemographic characteristics by group (no RT vs CXRT) and were compared using the Wilcoxon rank sum test, Fisher’s Exact test, and Pearson’s chi-squared test where appropriate. Univariable analysis and multivariable analysis (MVA) using Fine-Gray competing risk regression models were performed to identify risk factors associated with ED and EjD, where death was considered a competing risk. For the ED MVA, factors of CXRT treatment, age at cancer diagnosis, smoking history, dyslipidemia, depression, surgery, prostate cancer history, and tumor distance from anal verge were used, whereas for the MVA for EjD, only CXRT treatment and surgery were used due to limited events. We used the cumulative incidence function to estimate the cumulative incidence (CI) of ED and EjD with death as a competing risk. CIs were compared between the two treatment cohorts using Gray’s test. Time to ED and EjD were defined as the time between date of rectal cancer diagnosis and date of ED or EjD, respectively. Patients who did not experience the event of interest were censored at their date of last follow-up. The maximum rank statistic was used to investigate dosimetric values after which the risk of sexual dysfunction was greatest. These values were then introduced into competing risk regression models for sexual dysfunction. Overall survival (OS) was also estimated using the Kaplan-Meier method and compared between the two treatment groups with the log-rank test. Median follow-up time was calculated using the reverse Kaplan-Meier method and compared between the two groups. Descriptive statistics were used to describe the dose received by OARs implicated in sexual dysfunction in the cohort that received CXRT as part of LARC treatment.

## Results

### Patient population

Among 429 male patients diagnosed with LARC at age ≤50 years between 1995-2019, 350 received CXRT as part of their treatment and 79 were treated without RT (**Table 1**). Patients in the two groups were similar demographically with respect to age of diagnosis, year of diagnosis, race, and rectal cancer staging (p>0.05). However, patients in the CXRT group had lower-lying tumors than the no RT group (median distances from the anal verge of 7cm [IQR 4.5, 8.7] and 13cm [IQR 8.8, 18.0], respectively [p<0.001]). The groups were similar regarding risk factors for sexual dysfunction, such as body mass index, and histories of smoking, diabetes, hypertension, dyslipidemia, CAD, stroke, peripheral artery disease, depression, prostate enlargement (prostate cancer or benign prostatic hyperplasia), ADT use, renal disease, and ileostomy (p>0.05). The only risk factor that differed between the groups was alcohol use, which was higher in the no RT group (p=0.005). As expected, there were also higher rates of rectal cancer surgery in the no RT group versus the CXRT group (p<0.001).

**Table 1:** Patient demographics, risk factors, and treatment variables by treatment group.

Characteristic	No RT, N = 79^1^	CXRT, N = 350^1^	p-value^2^
**Age at Diagnosis**	46 (39, 49)	44 (38, 47)	0.063
**Year of Diagnosis**	2,013 (2,009, 2,015)	2,012 (2,005, 2,016)	0.13
**Race**			>0.9
African American	3 (3.8%)	18 (5.1%)	
Asian	9 (11%)	35 (10%)	
Other	1 (1.3%)	8 (2.3%)	
Unknown	2 (2.5%)	10 (2.9%)	
White	64 (81%)	279 (80%)	
**Ethnicity**			0.008
Unknown	5 (6.3%)	71 (20%)	
Hispanic or Latino	5 (6.3%)	29 (8.3%)	
Not Hispanic	69 (87%)	250 (71%)	
**Rectal Cancer AJCC Staging**			>0.9
2	13 (16%)	56 (16%)	
3	66 (84%)	294 (84%)	
**BMI**	26.9 (24.3, 29.8)	27.3 (24.5, 30.4)	0.6
**Smoking**			0.11
Any	19 (24%)	117 (33%)	
Never/Unknown	60 (76%)	233 (67%)	
**Alcohol**			0.005
Any	21 (27%)	48 (14%)	
None/Unknown	58 (73%)	302 (86%)	
**Diabetes**	5 (6.3%)	37 (11%)	0.3
**Hypertension**	22 (28%)	103 (29%)	0.8
**Dyslipidemia**	21 (27%)	81 (23%)	0.5
**CAD**	2 (2.5%)	17 (4.9%)	0.5
**Stroke or Precerebral Artery Occlusion**	0 (0%)	2 (0.6%)	>0.9
**Peripheral Artery Disease**	0 (0%)	1 (0.3%)	>0.9
**Depression**	7 (8.9%)	60 (17%)	0.066
Unknown	0	1	
**Prostate Cancer/BPH**	6 (7.6%)	27 (7.7%)	>0.9
**Androgen Deprivation Therapy**	0 (0%)	2 (7.7%)	>0.9
Unknown	73	324	
**Renal Disease**	4 (5.1%)	27 (7.7%)	0.4
**Ileostomy Status**			0.6
Never	30 (38%)	144 (41%)	
Prior but closed/Current	49 (62%)	206 (59%)	
**Surgery**			<0.001
LAR/Colectomy/Proctectomy/TAE/ABR	78 (99%)	262 (75%)	
None	1 (1.3%)	88 (25%)	
**Surgery Complication**	1 (1.3%)	5 (1.4%)	>0.9
**Median Tumor Distance from Anal Verge(cm)**	13.0 (8.8, 18.0)	7.0 (4.5, 8.7)	<0.001

1 Median (Q1, Q3); n (%).

2 Wilcoxon rank sum test; Pearson’s Chi-squared test; Fisher’s exact test.

### Erectile dysfunction

**Figure 1:**
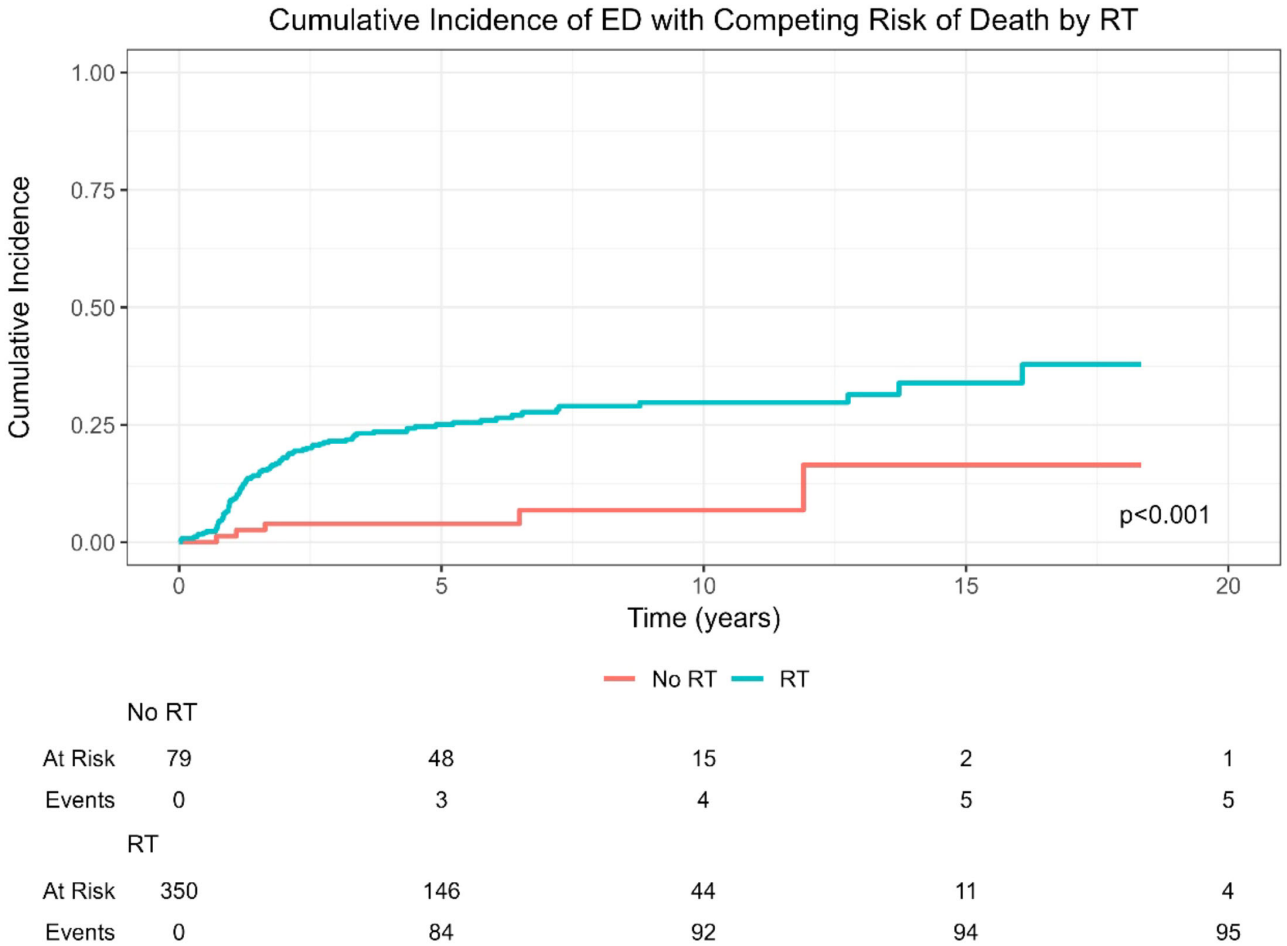
Cumulative incidence curves of ED by cohort with competing risk of death.

A total of 95 patients (27%) developed ED in the CXRT group and 5 patients (6%) developed ED in the no RT group. In the CXRT group, the 5- and 10-year cumulative incidences for ED were 25% (95% confidence interval [CI] 20-30%) and 30% (95%CI 24-35%), respectively. In the no RT group, the 5- and 10-year cumulative incidences for ED were 3.9% (95%CI 1.0-10%) and 6.8% (95%CI 1.9-16%), respectively (**Table 2**). The cumulative incidence of ED was statistically higher in the CXRT group vs the no RT group (Gray’s test p<0.001) (**Figure 1**).

**Table 2:** Cumulative incidence of ED and EjD with competing risk of death by cohort.

Characteristic	5-year	10-year	p-value^1^
**ED**			<0.001
No RT	3.9% (1.0%, 10%)	6.8% (1.9%, 16%)	
CXRT	25% (20%, 30%)	30% (24%, 35%)	
**EjD**			0.11
No RT	1.3% (0.11%, 6.2%)	1.3% (0.11%, 6.2%)	
CXRT	5.0% (3.0%, 7.7%)	7.0% (4.3%, 11%)	

1 Gray’s Test.

CXRT was significantly associated with ED on univariable analysis (**Table 3**) and maintained its significance as an independent risk factor for ED on MVA (Hazard Ratio [HR] 3.6 [95%CI 1.4, 9.8] p=0.011, N = 383). Risk factors such as depression (HR 2.1[95%CI 1.3, 3.2] p=0.001), smoking history (HR 1.56 [95%CI 1.1, 2.3] p=0.027) and CAD (HR 2.3 [95%CI 1.2, 4.6] p=0.019) were also associated with increased risk of ED univariably. Current or previous ostomy was also a risk factor for ED (HR 1.7 [95%CI 1.1, 2.6] p=0.019). No rectal cancer surgery (HR 0.48 [95%CI 0.26, 0.91] p=0.024) and higher tumor distance from the anal verge (HR 0.91 [95%CI 0.86, 0.96] p<0.001) were statistically protective against ED univariably. No other patient demographic or sexual dysfunction risk factor was associated with increased risk of ED.

**Table 3:** Univariable and multivariable competing risk regression models for ED with competing risk of death.

Characteristic	Univariable					Multivariable				
	N	Event N	HR^1^	95% CI^1^	p-value	N	Event N	HR^1^	95% CI^1^	p-value
**Radiation**	429	100				383	93			
No RT			—	—				—	—	
RT			4.68	1.92, 11.4	<0.001			3.63	1.35, 9.78	0.011
**Age at Diagnosis**	429	100	1.01	0.98, 1.05	0.5	383	93	1.02	0.98, 1.06	0.3
**Year of Diagnosis**	429	100	1.03	1.00, 1.06	0.027					
**Race**	429	100								
White			—	—						
African American			1.58	0.69, 3.59	0.3					
Asian			1.04	0.54, 2.02	0.9					
Other			0.50	0.07, 3.41	0.5					
Unknown			1.71	0.63, 4.67	0.3					
**Ethnicity**	429	100								
Not Hispanic			—	—						
Unknown			0.36	0.17, 0.73	0.005					
Hispanic or Latino			0.77	0.36, 1.65	0.5					
**Rectal Cancer AJCC Staging**	429	100								
3			—	—						
2			0.64	0.34, 1.22	0.2					
**Obese**	429	100	1.40	0.92, 2.12	0.12					
**Smoking**	429	100				383	93			
Never/Unknown			—	—				—	—	
Any			1.56	1.05, 2.30	0.027			1.33	0.87, 2.03	0.2
**Alcohol**	429	100								
None/Unknown			—	—						
Any			0.80	0.46, 1.41	0.5					
**Diabetes**	429	100	1.37	0.77, 2.44	0.3					
**Hypertension**	429	100	1.34	0.90, 2.00	0.2					
**Dyslipidemia**	429	100	1.41	0.93, 2.14	0.11	383	93	1.23	0.79, 1.92	0.4
**CAD**	429	100	2.30	1.15, 4.61	0.019					
**Depression**	428	100	2.07	1.34, 3.22	0.001	383	93	1.43	0.88, 2.34	0.2
**Prostate Cancer/BPH**	429	100	1.64	0.95, 2.83	0.078	383	93	1.40	0.79, 2.49	0.3
**Renal Disease**	429	100	1.72	0.89, 3.32	0.11					
**Ileostomy Status**	429	100								
Never			—	—						
Prior but closed/Current			1.67	1.09, 2.57	0.019					
**Concurrent Chemo**	429	100								
Chemo + Chemo RT			—	—						
No			1.78	0.82, 3.87	0.14					
Chemo RT			0.75	0.43, 1.29	0.3					
**Surgery**	429	100				383	93			
LAR/Colectomy/Proctectomy/TAE			—	—				—	—	
APR			0.56	0.17, 1.82	0.3			0.49	0.13, 1.80	0.3
None			0.48	0.26, 0.91	0.024			0.35	0.17, 0.72	0.005
**Surgery Complication**	429	100	2.09	0.86, 5.08	0.11					
**Tumor Distance from Anal Verge**	384	93	0.91	0.86, 0.96	<0.001	383	93	0.92	0.85, 0.99	0.027

1 HR = Hazard Ratio, CI = Confidence Interval.

### Ejaculatory dysfunction

There were 20 patients (6%) who developed EjD in the CXRT group compared to 1 patient in the no RT group (1%). The cumulative incidence of EjD was numerically higher in the CXRT group compared to no RT group (Gray’s test p=0.11). The 5- and 10-year cumulative incidences for EjD were 5% (95%CI 3-7.7%) and 7% (95%CI 4.3-11%), respectively, in the CXRT group, compared to 1.3% (95%CI 0.1-6.2%) at 5 and 10 years in the no RT group. None of the patients who developed EjD were using alpha blockers. Dyslipidemia and prior or current ostomy were associated with increased rates of EjD (p<0.05) (data not shown).

### Radiation dosimetry and technique

Median radiation doses by outcome are presented in **Supplementary Table 2**. The median prescription dose for patients who received IMRT was 5000cGy compared to a median prescription dose of 5040cGy in the 3D RT group. Numerically, doses to erectile tissue, penile bulb, and external genitalia were higher in patients who were diagnosed with ED. Univariably, IMRT was statistically associated with higher risk of ED compared to 3D RT (HR 1.53 [95%CI 1.02, 2.30], p=0.039) (**Supplementary Table 3**), however there was no difference in risk of EjD between 3D RT vs IMRT delivery. On MVA, factoring inguinal node treatment and dose to erectile tissue, IMRT was no longer a significant risk factor for ED (**Supplementary Table 3**). Similarly, on a sub-analysis of patients with tumors 0-8cm from the anal verge, IMRT was not statistically significant as a risk factor for ED compared to 3D RT (p=0.072) (**Table 4**). Differences in median maximum dose to OARs varied among 3D RT and IMRT techniques: median maximum dose to the penile bulb was 3720cGy for patients who received 3D RT and 4580cGy for those treated with IRMT, median maximum dose to the corpus cavernosum for 3D patients was 2350cGy and 2200cGy for IMRT patients, median maximum dose to corpus spongiosum was 3900cGy for patients receiving 3D RT and 4600cGy for patients receiving IMRT, and median maximum dose to the testes was 180cGy in 3D RT patients and 145cGy in IMRT patients.

**Table 4:** Univariable competing risk regression models for ED with competing risk of death, RT technique (CXRT patients with tumor distances 0-8.0cm from anal verge only).

Characteristic	N	Event N	HR^1^	95% CI^1^	p-value	q-value^2^
RT Technique						
3D	118	32	—	—		
IMRT/VMAT	103	36	1.54	0.96, 2.47	0.072	0.072

1 HR = Hazard Ratio, CI = Confidence Interval.

2 False discovery rate correction for multiple testing.

Dosimetric cutpoints were identified at which point a larger dose was associated with ED. These optimal cutpoints are displayed in **Table 5**. These cutpoints were evaluated on univariable analysis for ED (**Supplementary Table 3**).

**Table 5:** Optimal cutpoints of dosimetric variables identified by maximally selected rank statistic approach, CXRT patients only (n=182).

Variable	Optimal cutpoint
Delivered Fractions	25.0
RT Total Dose	5,040.0cGy
Corpus Cavernosum Dmax	2,544.9cGy
Corpus Cavernosum Dmean	779.9cGy
Corpus Cavernosum D1CC	2,461.3cGy
Corpus Cavernosum D3CC	1,381.1cGy
Corpus Cavernosum D5CC	1,316.9cGy
Corpus Spongiosum Dmax	4,800.2cGy
Corpus Spongiosum Dmean	466.7cGy
Corpus Spongiosum D1CC	4,659.2cGy
Corpus Spongiosum D3CC	3,260.2cGy
Corpus Spongiosum D5CC	2,692.2cGy
External Genitalia Dmean	102.7cGy
External Genitalia V20%	2.3%
External Genitalia V30%	0.0%
Penile Bulb Dmax	4,800.2cGy
Penile Bulb Dmean	3,496.3cGy
Penile Bulb D1CC	4,657.8cGy
Penile Bulb D3CC	4,185.2cGy
Penile Bulb D5CC	325.1cGy
Testes Dmax	99.4cGy
Glans Dmax	353.5cGy

Used in **Supplementary Table 3**.

Treatment of the inguinal nodes statistically increased the risk of ED within the CXRT group on MVA (HR 3.01 [95% CI 1.28, 7.1] p=0.012) (**Supplementary Table 3**). Total RT dose and increased maximum dose to the glans penis approached significance as a risk factor for EjD (p=0.07 for both). Otherwise, there was no statistically significant association between max dose or mean dose to the corpus cavernosum, corpus spongiosum, penile bulb, or testes and no significant association between the mean dose to the external genitalia and sexual dysfunction apart from what was mentioned previously (**Supplementary Table 4**).

### Follow-up and survival

The overall median follow-up was 6.3 years (95%CI 5.9, 7.2) with similar follow up times in the no RT group (5.8 years [95%CI 5.2, 7.8]) and CXRT group (6.6 years [95%CI 6.0, 7.4]) (p=0.3). Median OS was 25 years (95% CI 18, unreached) in our overall cohort. The overall 5-year OS was 83% (95%CI 79, 86%), with the 5-year OS in the no RT group being 90% (95%CI 83, 97%) and 81% (95%CI 77, 85%) in the CXRT group (p=0.013).

## Discussion

Understanding the risk of sexual dysfunction after pelvic RT is imperative as more men are being diagnosed with rectal cancer at a younger age and there is a greater emphasis on survivorship. Among 429 patients in our study, those treated with CXRT had a statistically higher cumulative incidence of ED compared to patients who were treated without RT. Although not statistically significant, there were numerically higher cumulative incidences of EjD at 5 and 10 years in patients receiving RT. Prior studies have demonstrated an increased risk of ED from pelvic radiation and lower-lying tumors (closer to the anal verge), which are consistent with our results (5, 8). Inguinal node treatment was also significantly associated with increased risk of ED in our study, and lower-lying rectal tumors that extend below the dentate line may require inguinal node coverage in radiation fields. Including the inguinal nodes may increase the dose of radiation to the genitalia, exacerbating the effect of RT on sexual dysfunction. Confounding the implication of RT contributing to ED is that previous studies have shown that lower surgical resections are also associated with higher risks of ED, which may be due to damage to the pelvic parasympathetic plexus (16) and 75% of patients in the CXRT group underwent surgery.

Considering that sexual dysfunction can be caused by vascular, neural, and psychological causes, medical comorbidities and surgery were considered. The groups were balanced in vascular and neurogenic risk factors. Psychological influences of depression and ostomy status were also balanced between both groups. However, despite a statistically higher rate of alcohol use and rectal cancer surgery in the no RT group, this group was less likely to have sexual dysfunction. Omission of surgery was found to be protective against ED in our study which correlates with surgery being a known risk factor for ED (17, 18). Although these studies also demonstrated surgery to be a cause of EjD, we did not see similar results in our study, which may be attributed to a lack of statistical power. Smoking is a well-established risk factor for ED through multiple mechanisms and our study also found smoking to be an independent risk factor in addition to RT on MVA.

In the overall cohort, IMRT was associated with a higher risk of ED compared to 3D RT despite similar prescription doses. Typically, pelvic IMRT is associated with less genitourinary toxicity compared to pelvic 3D RT because of better dose sparing of the OARs (19, 20); therefore, the increased risk of ED from IMRT may be due to increased scatter dose to the genitals. In our study, the median maximum dose to the penile bulb and corpus spongiosum was higher in patients who received IMRT, which may contribute to the association between IMRT and ED. However, the risk of ED from IMRT compared to 3D RT was no longer statistically higher when looking at a sub-analysis of patients with lower-lying tumors (≤8.0cm) (**Table 4**) or on MVA (**Supplementary Table 3**). This finding suggests that IMRT does not intrinsically increase risk, but instead there is likely preferential selection of IMRT over 3D RT in greater case complexity or in patients who are at increased baseline risk of toxicity (e.g., lower-lying tumors).

Recently, a phase 3 trial showed preoperative chemotherapy was noninferior to preoperative CXRT with respect to disease free survival for patients with LARC (21). This study also had higher patient reported sexual toxicities in the patients who received CXRT (22). These results combined with the results of the study presented here highlight the necessity and offer the opportunity to personalize treatments in the future. To help mitigate sexual toxicities in patients with prostate cancer, a study has shown that giving daily prophylactic sildenafil prior to RT start and through 6 months post treatment, can improve erectile function and overall sexual satisfaction at 12 months post RT (23). To date, a similar study has not been conducted for patients with rectal cancer; however, this approach should be investigated as a potential prophylactic mitigation strategy for patients who require CXRT treatment. Proton beam RT, with a near-zero exit dose, may also reduce sexual dysfunction compared to photon-based RT by reducing dose to certain OARs, such as the corporal bodies. Similarly, MRI-based planning may allow for better contouring of male genitalia and tissues involved in sexual function.

There are several limitations to this study that should be addressed. The study is limited by attrition at later time points which may introduce bias to the estimates of longer-term ED estimates at 5 years and 10 years as no formal imputation methods were employed for the missing outcome data. Additionally, the median follow-up time was slightly longer in the CXRT group compared to the no RT group which may contribute to surveillance bias. Patients undergoing CXRT typically have more visits with oncology providers compared to those treated without RT which could impact sexual dysfunction screening frequency and likelihood of capturing the event in our electronic medical records. In addition, we relied on diagnosis codes and referrals to our institution’s sexual health clinic to capture our primary outcomes, rather than a standardized baseline and post-treatment sexual health questionnaire, which may omit side effects that patients had addressed outside of our institution’s network and if they were already receiving treatment for sexual dysfunction. Since this was a retrospective study and our LARC patients were captured via diagnosis codes, low sigmoid and high rectal cancers are included in this cohort and may explain why the no RT group had significantly higher tumors compared to the CXRT group. Our OARs were also contoured on CT-based imaging rather than MRI-based, making anatomical delineation more challenging and increasing the element of human error. Further, our analysis only included dosimetry data for only 182 patients, which limited the statistical power and precluded multivariable modeling of dose-response relationships in this study. Of these, 50 patients had ED and 8 had EjD. This small number of events may have contributed to the lack of association between dose to OARs, which are known predictors of sexual dysfunction, and sexual dysfunction in our study, as shown in **Supplementary Table 3**. Despite these limitations, our data highlight the importance of larger future studies investigating sexual dysfunction in this patient population and the need to better capture dosimetric data related to these OARs. Future studies can expand on the dosimetric cutpoints provided in **Table 5** to further investigate clinically applicable dose constraints for minimizing risk of sexual dysfunction in this patient population. Given the limited sample size that hindered multivariable modeling or validation, the cutpoints provided here are exploratory and should not be used for treatment planning.

## Conclusion

Patients who received CXRT had a significantly higher cumulative incidence of ED compared to patients who received no RT. CXRT, depression, smoking, CAD, rectal cancer surgery, lower-lying tumors, and inguinal node treatment were all associated with increased risk of ED. The cumulative incidence of EjD was not statistically different in the patients who received CXRT compared to those without RT. This study suggests the need for future prospective studies aimed at reducing sexual dysfunction through patient-centered approaches such as reducing dose to the genitals via proton beam RT and MRI-based planning as well as using prophylactic sildenafil for patients requiring CXRT for LARC, as was done in patients with prostate cancer (23).

## Data Availability

The datasets presented in this article are not readily available because of data use agreements with the health system. Requests to access the datasets should be directed to Dr. Carla Hajj.

## Supplementary material

Supplementary Figure 1 Representative images showing organs at risk (OARs), gross tumor volume (GTV), planning target volume (PTV), clinical target volume (CTV) in patient who had inguinal node coverage in target volumes. OARs include corpus cavernosum (CC), corpus spongiosum (CS), glans penis (GL), penile bulb (PB), and testes.

Supplementary Table 1 Descriptive guide for contouring of OARs of interest. Table Citations (including web references): 1. https://www.ansci.wisc.edu/jjp1/ansci_repro/lab/lab2/boar_tract/crus_penis.html (Accessed March 17, 2025). 2. https://radiopaedia.org/articles/penis?lang=us (Accessed March 17, 2025). 3. Standring, Susan. Gray’s Anatomy. 40^th^ edn. Churchill Livingstone/Elsevier. 2008. ISBN: 9780443066849. 4. Dorland, W. Dorland’s Illustrated Medical Dictionary. 32nd edn. Philadelphia, USA: Elsevier Saunders. 2011. ISBN: 9781455726554. 5. Plants BA, Chen DT, Fiveash JB, Kim RY. Bulb of penis as a marker for prostatic apex in external beam radiotherapy of prostate cancer. Int J Radiat Oncol Biol Phys. 2003;56(4):1079-1084. doi:10.1016/s0360-3016(03)00116-0. 6. Kataria T, Gupta D, Goyal S, et al. Simple diagrammatic method to delineate male urethra in prostate cancer radiotherapy: an MRI based approach. Br J Radiol. 2016;89(1068):20160348. doi:10.1259/bjr.20160348. 7. Wallner KE, Merrick GS, Benson ML, Butler WM, Maki J, Tollenaar BG. Penile bulb imaging. Int J Radiat Oncol Biol Phys. 2002;53(4):928-933. doi:10.1016/s0360-3016(02)02805-5. 8. Brooks C, Hansen VN, Riddell A, Harris VA, Tait DM. Proposed genitalia contouring guidelines in anal cancer intensity-modulated radiotherapy. Br J Radiol. 2015;88(1051):20150032. doi:10.1259/bjr.20150032.

Supplementary Table 2 RT characteristics and dosimetric variables (median values, CXRT patients only).

Supplementary Table 3 Univariable competing risk models for ED with competing risk of death, dosimetric/RT binary variables (CXRT patients only).

Supplementary Table 4 Univariable competing risk regression models for ED with competing risk of death of dosimetric variables (CXRT patients only).
