# Supplementary material for: Quantifying the risk of sexual dysfunction in men treated with pelvic radiation therapy for locally advanced rectal cancer
Source: Front Oncol. 2026 Jun 17;16:1813135. doi: 10.3389/fonc.2026.1813135 (PMC13318684; doi:10.3389/fonc.2026.1813135)
Supplement: Supplementary Figure 1 — Representative images showing organs at risk (OARs), gross tumor volume (GTV), planning target volume (PTV), clinical target volume (CTV) in patient who had inguinal node coverage in target volumes. OARs include corpus cavernosum (CC), corpus spongiosum (CS), glans penis (GL), penile bulb (PB), and testes. [file DataSheet1.pdf]

Supplemental Table 1: Descriptive Guide for Contouring of OARs of Interest

Organ	Description
Corpus Cavernosum	Contouring begins at the base of the penis where the two corpora converge, identified as two parallel, cylindrical structures in the penile shaft dorsal to the corpus spongiosum. From this convergence point, the contouring extends along the penile shaft to just before the glans penis.[1,2,3]
Corpus Spongiosum	Contouring begins at the penile bulb and continues through the entire length of the penile shaft. The corpus spongiosum is identified as a single cylindrical structure ventral to the corpus cavernosa, which extends distally to encompass the glans penis.[2,3,4]
Glans Penis	Contoured as the bulbous distal expansion of the corpus spongiosum, forming the tip of the penis.[2,3]
Penile Bulb	Contouring begins at the inferior surface of the urogenital diaphragm. The oval-shaped contour extends inferiorly and narrows anteriorly as the proximal part of the corpus spongiosum without continuing past the penoscrotal junction into the penile shaft.[5,6,7]
Testes	Contour encompasses both testes within the scrotal sac.[3]
External Genitalia	Contouring begins at the level of the pubic symphysis, including skin and superficial tissues anterior to it. The contour extends laterally to include the inguinal creases, inferiorly to encompass the scrotum and comprises the visible external penile structures.[3,8]

Table Citations (including web references):

1. https://www.ansci.wisc.edu/jjp1/ansci_repro/lab/lab2/boar_tract/crus_penis.html (Accessed March 17, 2025)
2. https://radiopaedia.org/articles/penis?lang=us (Accessed March 17, 2025)
3. Standring, Susan. Gray's Anatomy. 40th edn. Churchill Livingstone/Elsevier. 2008. ISBN: 9780443066849
4. Dorland, W. Dorland's Illustrated Medical Dictionary. 32nd edn. Philadelphia, USA: Elsevier Saunders. 2011. ISBN: 9781455726554
5. Plants BA, Chen DT, Fiveash JB, Kim RY. Bulb of penis as a marker for prostatic apex in external beam radiotherapy of prostate cancer. Int J Radiat Oncol Biol Phys. 2003;56(4):1079-1084. doi:10.1016/s0360-3016(03)00116-0
6. Kataria T, Gupta D, Goyal S, et al. Simple diagrammatic method to delineate male urethra in prostate cancer radiotherapy: an MRI based approach. Br J Radiol. 2016;89(1068):20160348. doi:10.1259/bjr.20160348
7. Wallner KE, Merrick GS, Benson ML, Butler WM, Maki J, Tollenaar BG. Penile bulb imaging. Int J Radiat Oncol Biol Phys. 2002;53(4):928-933. doi:10.1016/s0360-3016(02)02805-5
8. Brooks C, Hansen VN, Riddell A, Harris VA, Tait DM. Proposed genitalia contouring guidelines in anal cancer intensity-modulated radiotherapy. Br J Radiol. 2015;88(1051):20150032. doi:10.1259/bjr.20150032

Supplemental Figure 1: Representative images showing organs at risk (OARs), gross tumor volume (GTV), planning target volume (PTV), clinical target volume (CTV) in patient who had inguinal node coverage in target volumes. OARs include corpus cavernosum (CC), corpus spongiosum (CS), glans penis (GL), penile bulb (PB), and testes.

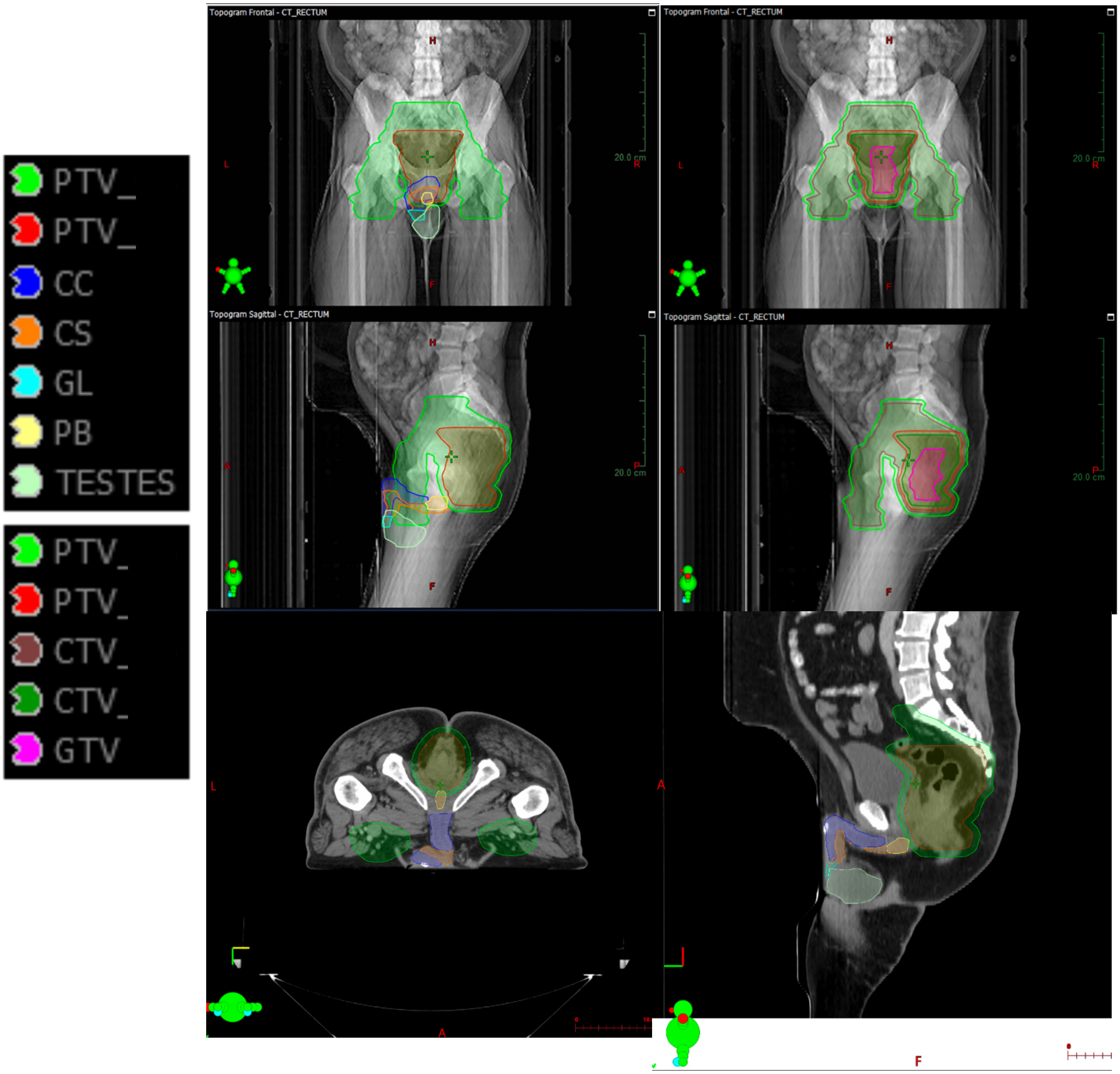

Supplement Table 2: RT Characteristics and Dosimetric Variables (Median Values, CXRT Patients Only)

	Overall		ED			Ejaculatory Dysfunction		
**Characteristic**	**N**	**N = 350**[1]	**N**	**No ED** N = 255[1]	**ED** N = 95[1]	**N**	**No Ejaculatory Dysfunction** N = 330[1]	**Ejaculatory Dysfunction** N = 20[1]
**RT Technique**	350		350			350		
3D		196 (56%)		148 (58%)	48 (51%)		188 (57%)	8 (40%)
IMRT/VMAT		143 (41%)		98 (38%)	45 (47%)		131 (40%)	12 (60%)
unknown		11 (3.1%)		9 (3.5%)	2 (2.1%)		11 (3.3%)	0 (0%)
**Characteristic**	**N**	**N = 288**[1]	**N**	**No ED** N = 208[1]	**ED** N = 80[1]	**N**	**No Ejaculatory Dysfunction** N = 272[1]	**Ejaculatory Dysfunction** N = 16[1]
**Delivered Fractions**	288	28 (5, 31)	288	28 (5, 31)	26 (16, 30)	288	28 (5, 31)	25 (16, 28)
**RT Total Dose**	288	5,040 (1,200, 5,600)	288	5,040 (1,200, 5,580)	5,020 (2,880, 5,600)	288	5,040 (1,200, 5,600)	5,000 (2,880, 5,040)
**Characteristic**	**N**	**N = 182**[1]	**N**	**No ED** N = 132[1]	**ED** N = 50[1]	**N**	**No Ejaculatory Dysfunction** N = 174[1]	**Ejaculatory Dysfunction** N = 8[1]
**Corpus Cavernosum Dmax**	182	2,247 (126, 5,821)	182	2,146 (140, 5,821)	2,766 (126, 5,238)	182	2,259 (140, 5,821)	1,858 (126, 4,584)
**Corpus Cavernosum Dmean**	182	907 (68, 3,103)	182	894 (91, 2,792)	1,034 (68, 3,103)	182	920 (91, 3,103)	583 (68, 2,339)
**Corpus Cavernosum D1cc**	182	1,898 (104, 5,680)	182	1,775 (129, 5,680)	2,269 (104, 5,007)	182	1,923 (129, 5,680)	1,523 (104, 4,279)
**Corpus Cavernosum D3cc**	182	1,585 (95, 4,863)	182	1,519 (122, 4,863)	1,989 (95, 4,521)	182	1,592 (122, 4,863)	1,397 (95, 3,936)
**Corpus Cavernosum D5cc**	182	1,492 (89, 4,649)	182	1,362 (117, 4,649)	1,604 (89, 4,079)	182	1,520 (117, 4,649)	1,303 (89, 3,785)
**Corpus Spongiosum Dmax**	182	4,252 (191, 5,707)	182	4,162 (191, 5,707)	4,519 (286, 5,426)	182	4,282 (191, 5,707)	2,176 (402, 5,157)
**Corpus Spongiosum Dmean**	182	858 (61, 3,224)	182	836 (61, 3,224)	952 (89, 3,223)	182	866 (61, 3,224)	422 (89, 1,970)
**Corpus Spongiosum D1cc**	182	3,128 (175, 5,503)	182	2,950 (175, 5,503)	4,103 (250, 5,213)	182	3,325 (175, 5,503)	1,677 (250, 5,009)

	Overall		ED			Ejaculatory Dysfunction		
**Characteristic**	**N**	**N = 350**[1]	**N**	**No ED** N = 255[1]	**ED** N = 95[1]	**N**	**No Ejaculatory Dysfunction** N = 330[1]	**Ejaculatory Dysfunction** N = 20[1]
**Corpus Spongiosum D3cc**	182	2,089 (154, 5,309)	182	1,915 (154, 5,309)	2,522 (197, 5,143)	182	2,157 (154, 5,309)	1,128 (197, 4,625)
**Corpus Spongiosum D5cc**	182	1,660 (111, 5,278)	182	1,584 (111, 5,278)	2,180 (169, 5,133)	182	1,707 (111, 5,278)	831 (169, 3,985)
**External Genitalia Dmean**	182	363 (0, 2,268)	182	330 (0, 1,607)	418 (44, 2,268)	182	369 (0, 2,268)	239 (44, 1,315)
**External Genitalia V20%**	182	0 (0, 1,632)	182	0 (0, 45)	0 (0, 1,632)	182	0 (0, 1,632)	0 (0, 30)
**External Genitalia V30%**	182	0.00 (0.00, 1,598.50)	182	0.00 (0.00, 20.20)	0.00 (0.00, 1,598.50)	182	0.00 (0.00, 1,598.50)	0.00 (0.00, 20.20)
**Glands Dmax**	182	854 (9, 4,578)	182	807 (9, 4,578)	894 (30, 4,422)	182	869 (9, 4,578)	419 (85, 1,529)
**Penile Bulb Dmax**	182	4,282 (174, 5,821)	182	4,162 (174, 5,821)	4,519 (181, 5,426)	182	4,308 (174, 5,821)	2,048 (181, 5,157)
**Penile Bulb Dmean**	182	2,176 (93, 5,237)	182	2,115 (121, 5,237)	2,526 (93, 5,125)	182	2,196 (121, 5,237)	885 (93, 4,474)
**Penile Bulb D1cc**	182	3,128 (156, 9,736)	182	2,961 (156, 9,736)	4,033 (156, 5,212)	182	3,245 (156, 9,736)	1,356 (156, 5,010)
**Penile Bulb D3cc**	182	1,923 (132, 5,310)	182	1,809 (132, 5,310)	2,465 (137, 5,132)	182	2,065 (132, 5,310)	812 (137, 4,627)
**Penile Bulb D5cc**	182	1,383 (114, 5,278)	182	1,232 (114, 5,278)	1,987 (125, 5,111)	182	1,469 (114, 5,278)	606 (125, 3,937)
**Testes Dmax**	182	158 (25, 5,088)	182	154 (25, 4,673)	165 (29, 5,088)	182	163 (25, 5,088)	121 (29, 620)
**Inguinals**	182	33 (18%)	182	18 (14%)	15 (30%)	182	30 (17%)	3 (38%)

[1]n (%); Median (Min, Max)

Supplemental Table 3: Univariable Competing Risk Models for ED with competing risk of death, Dosimetric/RT Binary Variables (CXRT patients only)

Characteristic	N	HR[1]	95% CI[1]	p-value
**Delivered Fractions(Binary)**	288			
<= 25		—	—	
> 25		0.66	0.43, 1.02	0.060
**RT Total Dose(Binary)**	288			
<= 5040		—	—	
> 5040		0.46	0.19, 1.13	0.089
**Corpus Cavernosum Dmax(Binary)**	182			
<= 2544.9		—	—	
> 2544.9		1.77	1.02, 3.09	0.043
**Corpus Cavernosum Dmean(Binary)**	182			
<= 779.9		—	—	
> 779.9		1.56	0.87, 2.79	0.13
**Corpus Cavernosum D1cc(Binary)**	182			
<= 2461.3		—	—	
> 2461.3		1.77	1.02, 3.07	0.043
**Corpus Cavernosum D3cc(Binary)**	182			
<= 1381.1		—	—	
> 1381.1		2.06	1.10, 3.86	0.025
**Corpus Cavernosum D5cc(Binary)**	182			
<= 1316.9		—	—	
> 1316.9		2.16	1.17, 3.98	0.014
**Corpus Spongiosum Dmax(Binary)**	182			
<= 4800.2		—	—	
> 4800.2		1.98	1.13, 3.46	0.017
**Corpus Spongiosum Dmean(Binary)**	182			
<= 466.7		—	—	
> 466.7		1.87	0.95, 3.65	0.068
**Corpus Spongiosum D1cc(Binary)**	182			
<= 4659.2		—	—	
> 4659.2		2.05	1.14, 3.70	0.017
**Corpus Spongiosum D3cc(Binary)**	182			
<= 3260.2		—	—	
> 3260.2		1.76	1.01, 3.07	0.045
**Corpus Spongiosum D5cc(Binary)**	182			
<= 2692.2		—	—	
> 2692.2		1.87	1.07, 3.27	0.027
**External Genitalia Dmean(Binary)**	182			
<= 102.7		—	—	
> 102.7		3.03	0.95, 9.61	0.060
**External Genitalia V20%(Binary)**	182			
<= 2.3		—	—	
> 2.3		2.18	1.24, 3.85	0.007
**External Genitalia V30%(Binary)**	182			
<= 0		—	—	
> 0		2.38	1.27, 4.47	0.007

**Characteristic**	**N**	**HR**[1]	**95% CI**[1]	**p-value**
**Penile Bulb Dmax(Binary)**	182			
<= 4800.2		—	—	
> 4800.2		2.00	1.15, 3.48	0.015
**Penile Bulb Dmean(Binary)**	182			
<= 3496.3		—	—	
> 3496.3		1.70	0.97, 2.96	0.063
**Penile Bulb D1cc(Binary)**	182			
<= 4657.8		—	—	
> 4657.8		2.01	1.13, 3.59	0.018
**Penile Bulb D3cc(Binary)**	182			
<= 4185.2		—	—	
> 4185.2		1.93	1.06, 3.53	0.031
**Penile Bulb D5cc(Binary)**	182			
<= 325.1		—	—	
> 325.1		2.66	1.05, 6.77	0.040
**Testes Dmax (Binary)**	182			
<= 99.4		—	—	
> 99.4		2.28	1.13, 4.60	0.022
**Glands Dmax(Binary)**	182			
<= 353.5		—	—	
> 353.5		1.66	0.91, 3.02	0.10

[1]HR = Hazard Ratio, CI = Confidence Interval

Supplemental Table 4: Univariable Competing Risk Regression Models for ED with Competing Risk of Death of Dosimetric Variables (CXRT patients only)

	Univariable					Multivariable				
**Characteristic**	**N**	**Event N**	**HR[1]**	**95% CI[1]**	**p-value**	**N**	**Event N**	**HR[1]**	**95% CI[1]**	**p-value**
**RT Technique**	339	93				182	50			
3D			—	—				—	—	
IMRT/VMAT			1.53	1.02, 2.30	0.039			0.98	0.48, 2.01	>0.9
**Delivered Fractions**	288	80	0.99	0.94, 1.05	0.8					
**RT Total Dose**	288	80	1.00	1.00, 1.00	0.8					
**Corpus Cavernosum Dmax**	182	50	1.00	1.00, 1.00	0.2	182	50	1.00	1.00, 1.00	0.7
**Corpus Cavernosum Dmax EQD2**	182	50	1.01	0.99, 1.03	0.2					
**Corpus Cavernosum Dmean**	182	50	1.00	1.00, 1.00	0.3	182	50	1.00	1.00, 1.00	0.9
**Corpus Cavernosum Dmean EQD2**	182	50	1.02	0.98, 1.07	0.3					
**Corpus Spongiosum Dmax**	182	50	1.00	1.00, 1.00	0.3	182	50	1.00	1.00, 1.00	0.5
**Corpus Spongiosum Dmean EQD2**	182	50	1.01	0.99, 1.03	0.2					
**Corpus Spongiosum Dmean**	182	50	1.00	1.00, 1.00	0.4	182	50	1.00	1.00, 1.00	0.3
**Corpus Spongiosum Dmean EQD2**	182	50	1.02	0.98, 1.06	0.4					
**External Genitalia Dmean**	182	50	1.00	1.00, 1.00	0.4					
**External Genitalia Dmean EQD2**	182	50	1.04	0.94, 1.15	0.4					
**Glands Dmax**	182	50	1.00	1.00, 1.00	0.5					
**Glands Dmax EQD2**	182	50	1.01	0.98, 1.04	0.5					
**Penile Bulb Dmax**	182	50	1.00	1.00, 1.00	0.3					
**Penile Bulb Dmax EQD2**	182	50	1.01	0.99, 1.03	0.2					
**Penile Bulb Dmean**	182	50	1.00	1.00, 1.00	0.2					
**Penile Bulb Dmean EQD2**	182	50	1.01	0.99, 1.03	0.2					
**Testes Dmax**	182	50	1.00	1.00, 1.00	>0.9					
**Testes Dmax EQD2**	182	50	1.01	0.96, 1.06	0.8					
**Inguinals**	182	50	2.38	1.30, 4.34	0.005	182	50	3.01	1.28, 7.10	0.012

[1]HR = Hazard Ratio, CI = Confidence Interval

[2]False discovery rate correction for multiple testing
